# Farmers’ Intended Weed Management after a Potential Glyphosate Ban in Austria

**DOI:** 10.1007/s00267-022-01611-0

**Published:** 2022-02-25

**Authors:** Teresa Matousek, Hermine Mitter, Bernadette Kropf, Erwin Schmid, Stefan Vogel

**Affiliations:** Institute for Sustainable Economic Development, Department of Economics and Social Sciences, University of Natural Resources and Life Sciences, Vienna (BOKU), Feistmantelstraße 4, 1180 Vienna, Austria

**Keywords:** Pest control, Crop protection, Qualitative analysis, Behavioral intention, Glyphosate-based herbicide

## Abstract

Glyphosate is controversially discussed because of its alleged harmful effects on human health and the environment. Although it is approved until December 2022 in the European Union, the Austrian government discusses a national ban. Research on farmers’ intentions to deal with upcoming pesticide policy changes is limited and planned responses to a national glyphosate ban may inform accompanying measures and the development of weed management alternatives. Therefore, we have conducted 41 qualitative semi-structured interviews with farmers to explore their intended weed management if glyphosate-based herbicides were no longer available in Austria. The interviews were systematically analyzed, whereby the Theory of Planned Behavior (TPB) with its three social-psychological constructs served as guidance, i.e., attitude toward the planned behavior, subjective norm, and perceived behavioral control toward the planned behavior. We grouped farmers based on differences in their behavioral intentions toward glyphosate-free weed management, and identified four types of farmers by assigning group-specific attributes of the TPB constructs to the groups of farmers with similar behavioral intentions. Given a national glyphosate ban, the farmers intend to implement either mechanical or chemical alternatives, which would be solely applied or combined with changes in cultivation. Attitude toward the planned behavior, descriptive norms, and perceived behavioral control affect behavioral intentions, whereas injunctive norms do not differ much between the interviewed farmers. What unites the four types of farmers is that they would rather accept a glyphosate ban, if weed management alternatives with similar effectiveness and costs were available.

## Introduction

Today, more than 30% of crop yields are lost due to insect pests, plant pathogens, and weeds (hereafter collectively termed “pests”) even though the amount of chemical pesticides applied in agriculture has risen since the Green Revolution (Riegler 2018). Chemical pesticides embrace a variety of compounds, whereby herbicides constitute the majority of globally applied pesticides (Gressel 2011). Ideally, a chemical pesticide is lethal to the targeted pest without harming non-targeted species including humans. However, pesticide residues have been repeatedly detected in food products (e.g., Medina‐Pastor and Triacchini 2020) and the environment (e.g., Silva et al. 2019) and have thus increased human and environmental exposure to pesticides (Sharma et al. 2020). Even if the concentrations or levels are often below predictions or legal standards (Medina‐Pastor and Triacchini 2020; Silva et al. 2019) public awareness of and concern about human health and environmental risks resulting from chemical pesticide use have substantially increased (Kudsk and Mathiassen 2020; Moser and Dondi 2016). These developments have culminated in research initiatives toward chemical pesticide-free agriculture (INRAE 2020) and public policies to foster integrated pest management and organic farming (Lefebvre et al. 2015).

Pesticide policies aim to reduce negative externalities of farmers’ pest management. Their design and implementation differs by country or supra-national entity, which has become particularly conspicuous during the renewal of approval of the active substance glyphosate. Glyphosate is the basis of various non-selective herbicide formulations that target a broad range of broadleaf weeds and grasses. The approval of glyphosate and other active substances is based on an established evaluation procedure for a maximum of ten years in the European Union (EU), whereas formulations and applications are authorized on the national level (Regulation No 1107/2009). Glyphosate is approved in the EU until December 2022, and an application for the renewal of approval beyond 2022 was submitted in December 2019. By contrast, registered pesticides are reviewed every 15 years in the United States (U.S.), and the U.S. Environmental Protection Agency released an interim decision for glyphosate confirming that it satisfies the statutory standard for registration in 2020 (US-EPA 2020).

Given these different procedures and the wide application of glyphosate-based herbicides globally (Maggi et al. 2019), glyphosate has probably been the most intensively discussed active substance for years (Kudsk and Mathiassen 2020). On the one hand, it is valued for its non-selectiveness, effectiveness, ease of use, environmental compatibility, and low cost (Baylis 2000; Duke and Powles 2008). On the other hand, concerns were raised with respect to its potentially adverse effects on human health and the environment. For instance, the International Agency for Research and Cancer (IARC 2017) classified glyphosate as “probably carcinogenic to humans,” based on hazard assessment. However, this conclusion was not supported by risk assessments (considering also exposure) conducted by a number of regulatory authorities. After an extensive scientific review, Health Canada (2019) even noted that “No pesticide regulatory authority in the world currently considers glyphosate to be a cancer risk to humans at the levels at which humans are currently exposed.”

The various reports and assessments stimulated controversial discussions about the active substance glyphosate in Austria. The strong opponency among European (European Commission 2017) and Austrian citizens caused the Austrian parliament to ban glyphosate by law in 2019 (Nationalrat 2019), despite experts had raised the non-conformity with European law (Damjanovic and Eisenberger 2019; Obwexer 2017). The national law did not become operative due to formal errors during the notification process. However, the Austrian parliament re-initiated the discussion on national law to ban glyphosate in 2020. Even if active substances are approved at the European level, member states can restrict the use of glyphosate-based herbicides, for instance, in ecologically sensitive areas or through limiting rates, periods and fields of application (Damjanovic and Eisenberger 2019).

The uncertain future of glyphosate-based herbicides in Austria may confuse or bother farmers, and may affect farmers’ weed management. Knowledge about farmers’ behavioral intention is crucial for the success of pesticide policies and the development of accompanying measures (Buchholz and Musshoff 2021; Dessart et al. 2019). Hence, a better understanding of farmers’ intended weed management is of relevance.

Previous research has focused on quantitative assessments of potential economic outcomes of a glyphosate ban. For instance, Brookes et al. (2017) estimated annual reductions in global farm income gains to be higher than US$6 billion if restrictions in glyphosate use result in a reversion to conventional crops (i.e., away from genetically modified herbicide-tolerant crops). In the European context, where genetically modified crops are not approved, economic assessments of a potential glyphosate ban point to additional variable costs (e.g., for machinery, fuel and labor; Mal et al. 2015) and to reductions in yield (e.g., due to higher weed pressure; Cook et al. 2010; Schmitz and Garvert 2012). The assessments also emphasize that economic outcomes of replacing glyphosate-based herbicides in arable farming largely depend on natural (e.g., soil quality, slope and climate) and farm conditions (e.g., machinery equipment and crop rotation system; Kehlenbeck et al. 2016; Steinmann et al. 2012).

Farmers’ planned responses to changes in pesticide policies have received limited attention. So far, factors that constrain farmers or their intention to reduce pesticide inputs have been addressed including farmers’ risk perception and preferences (Bakker et al. 2021; Möhring et al. 2020), farmers’ subjective norms (Bakker et al. 2021), farmers’ perceived limited capacity and autonomy (Bakker et al. 2021), farmers’ disbelief about human health and environmental risks from chemical pesticides (Lichtenberg and Zimmerman 1999), biased heuristics and habit (Perry et al. 2019), confined cooperation between researchers, extension services and farmers (Lamichhane et al. 2016), ecological illiteracy (Wyckhuys et al. 2019), and unfavorable farm characteristics and production conditions (Andert et al. 2015; Qin and Lü 2020; Wiese et al. 2018).

However, little is known about how farmers may deal with new pesticide policies or expected policy changes, and it remains unclear which attitudes, subjective norms, and farmers’ perceived facilitating and impeding factors shape their intentions to adjust their pest management to such policies. The need to improve the understanding of factors influencing the adoption of weed management alternatives has been emphasized as a research priority to successfully manage agricultural pests (Lamichhane et al. 2017). With respect to glyphosate, farmers have been asked about their current application patterns and rates (Givens et al. 2009; Perry et al. 2019; Wiese et al. 2018) and their experiences with glyphosate-resistant weeds (Foresman and Glasgow 2008), but rarely about replacement strategies if glyphosate-based herbicides were no longer available. One exception is a structured survey among German farmers that found that farmers expect an increase in soil cultivation intensity and, on average, one additional application of a post-emergence herbicide for specific crops, if glyphosate-based herbicides were no longer available (Steinmann et al. 2012). However, this study did not report on farmers’ attitudes toward the weed management alternatives, their subjective norms and their capacity to act.

We apply a qualitative research approach to explore how farmers who currently use glyphosate-based herbicides on their farms intend to respond to a national glyphosate ban that is constantly discussed. In spite of a potential EU-wide change in pesticide policies, the focus of the study is Austria for two reasons. First, ongoing discussions on a national glyphosate ban combined with considerable efforts to implement a national law (though failed in its first attempt) increase uncertainties and pressure on Austrian farmers to familiarize with weed management alternatives. Second, Austria is characterized by a high diversity of farm types and production conditions that may affect farmers’ response strategies to a national glyphosate ban.

We aim to improve our understanding of farmers’ intended weed management if a national glyphosate ban became effective in Austria. The Theory of Planned Behavior (TPB; Ajzen 1985, 1991, 2012) guides our empirical analysis. This theory considers behavioral intention as a direct determinant of behavior, in terms of behavioral motivation. Behavioral intention is formed from three predictors, (i) from attitudes toward the behavior in question, (ii) from subjective norm, and (iii) from perceived behavioral control, which consists of assumptions about how easy or difficult it is for the subject to perform the behavior (Ajzen 1985, 1991). The definition and character of predictors makes the theory attractive to guide research on reasons for behavioral intention based on both quantitative and qualitative methods.

The TPB has already been used in pesticide research to identify determinants of farmers’ intentions to reduce their pesticide use and environmental harm (Bakker et al. 2021) and to apply integrated pest management (Despotović et al. 2019; Rezaei et al. 2019). While the TPB is mostly employed for quantitative analyses, its usefulness for mixed-method (Bijttebier et al. 2018) and qualitative research approaches has also been demonstrated (Hall et al. 2019; Ranjan et al. 2019; Renzi and Klobas 2008). Furthermore, the importance of qualitative, social-psychological analyses has been stressed in order to increase our knowledge of behavioral patterns beyond opportunism (Mann 2018; Wiese et al. 2018).

The article is structured as follows. In “Material and methods,” we describe the theoretical framework and the methods applied. In “Farmers’ intended weed management after a national glyphosate ban,” we present results from our qualitative research that are put in the research context in “Discussion.” We close the article with “Conclusions”.

## Material and Methods

### Case Study of Austria

In Austria, about 9% (114,500 ha) of total cropland are treated with glyphosate-based herbicides, mostly for maize, sugar beet, soybean and potatoes (pre-sowing, often in combination with conservation tillage), and cereals (post-harvesting). Glyphosate-based herbicides are also used in orchards on about 12% of the total area (i.e., 5000 ha) and in vineyards on about 60% of the total area (i.e., 25,000 ha). Furthermore, they are sometimes applied for cultivating vegetables and Christmas trees, on grassland, and in forestry (Besenhofer 2019). In total, 242 t of glyphosate were sold in Austria in 2018, with a decreasing trend over the last 10 years (AGES 2020). This trend can be partly ascribed to the increase in organic farming (BMLRT 2019), and the Austrian agri-environmental program which supports the renouncement of chemical herbicides (BMLRT 2016). In Austria, regulations for glyphosate-based herbicides are already stricter than in most other EU member states because the application for the acceleration of ripeness (desiccation) is legally prohibited (Pflanzenschutzmittelgesetz 2011). As mentioned above, the Austrian parliament is discussing a national law to ban glyphosate, after its first draft law did not come into force.

### Theoretical Framework

The TPB (Ajzen 1985, 1991, 2012) guides our empirical research because of four reasons. First, the usefulness of the TPB to explain behavioral intention (i.e., planned behavior) has been proven in various behavioral domains (e.g., Armitage and Conner 1999), including pest management (Bakker et al. 2021; Despotović et al. 2019; Rezaei et al. 2019). Second, the TPB is parsimonious in the theoretical constructs considered and, at the same time, sufficiently broad to capture the determinants of behavioral intentions. As such, it can support the design of the interview guide (section “Developing the interview materials”), the deductively driven research phase as well as structured thinking and analyses of the behavioral intention in question (Ajzen 2020). Third, behavioral intention is considered the “immediate antecedent of behavior” in the TPB, i.e., the likelihood to perform the behavior in question increases with a stronger intention. In the case of a regulation not yet implemented (such as the discussed national glyphosate ban), the investigated behavioral intentions provide relevant insights into how individuals may respond (Ajzen 1991, 2020). Fourth, the TPB can be applied in choice situations such that the behavioral alternative revealing the strongest intention will finally be chosen (Ajzen 2020; Ajzen and Fishbein 1969). This is meaningful in the context of pest management where different types of control measures are available (e.g., Kropf et al. 2020) and in situations where conformity to the law requires action.

In the empirical analysis, we refer to the three social-psychological constructs of the TPB that determine an individual’s behavioral intention in a specific context, i.e., attitude toward the planned behavior, subjective norm, and perceived behavioral control toward the planned behavior (Ajzen 1985, 1991). We define the behavioral intention in question as Austrian farmers’ weed management (action) without glyphosate-based herbicides (target) on-farm (context) after a national glyphosate ban (time). Attitude toward the planned behavior is defined as the degree to which an individual thinks about the performance of the behavior as favorable or unfavorable. It is determined by the set of accessible beliefs about the behavior’s likely outcomes or experiences. Subjective norm is the perceived social pressure and is composed of injunctive (if other relevant people approve the behavior) and descriptive (how other relevant people behave) normative beliefs (Fishbein and Ajzen 2010). Perceived behavioral control summarizes the perceived facilitating and impeding factors to perform the behavior in question (Ajzen 1991). Both, a positive attitude and a positive subjective norm generate motivation toward the behavior. However, the perceived behavioral control needs to be sufficiently strong to trigger action. Finally, it can be assumed that background factors such as age, education, personality or knowledge affect intentions and behavior indirectly via behavioral, normative or control beliefs. Therefore, if considered promising, background factors can also be examined (Ajzen 2020).

A major impetus for the development of the TPB in the 1970s was the realization that traditional measures of mostly very general attitudes could not predict specific behavior because a variety of different behaviors could be associated with such general attitudes (Fishbein and Ajzen 1974, 1975; van Liere and Dunlap 1981; Weigel et al. 1974). Based on these findings on the attitude–behavior relationship, Fishbein and Ajzen (1975) suggested that both attitudes and behavior should be measured at the same level of specificity or generality (the principle of compatibility; Ajzen 1988, 2005). However, the principle of compatibility entails that the measures of attitudes eventually proposed in the TPB represent planning structures rather than attitudes, which is the most relevant criticism of this theory (Eckes and Six 1994). The behavioral intention in the TPB is, thus, no longer an attitude, but a measure of behavioral actualization in the sense of an expectation for the occurrence of one’s behavior (Vogel 1999). This point of criticism does not apply to the application of the TPB in this analysis for two reasons. First, we do not specify the attitudes in advance, which is in accordance with the qualitative methodological approach of this research. Second, we record the a priori behavioral intentions (i.e., planned behavior), but do not link them as a determinant with ex post collected actual behavior as a dependent variable of the behavioral intention. This would only be possible by observing the behavior after a potential glyphosate ban.

### Research Questions

The Austrian government is constantly discussing a national law to ban the active substance glyphosate. Accordingly, farmers who currently apply glyphosate-based herbicides face uncertainties and may be concerned or worried about their future weed management. With our research, we aim to explore Austrian farmers’ perceptions and intentions to respond to a potential national glyphosate ban. Knowledge about farmers’ weed management intentions can support the design of accompanying measures and, hence, contribute to the effectiveness of pesticide policies. In detail, we address the following five research questions:

(i) What are the intentions of farmers, who apply glyphosate-based herbicides, toward their weed management after a potential glyphosate ban in Austria? In particular, which weed management alternatives do farmers plan for a period after a national glyphosate ban?
(ii) Which attitudes toward the behavior influence farmers’ behavioral intentions, i.e., weed management intentions?
(iii) Which subjective norm do farmers perceive with respect to their behavioral intentions? In particular, which injunctive and descriptive normative beliefs do farmers hold?
(iv) Which facilitating and impeding factors do farmers perceive to perform their behavioral intentions (i.e., perceived behavioral control)?
(v) Which groups of farmers can be identified based on differences in their behavioral intentions, and, if applicable, by assigning group-specific attributes of attitudes toward the behavior, subjective norm and perceived behavioral control toward the behavior, which types of farmers can be identified?

### Developing the Interview Materials

We developed an interview guide based on the theoretical constructs of the TPB and a comprehensive literature review. The literature review focused on pest management and glyphosate use in agriculture, on attitudes and farmers’ perceptions of social factors and their capacity underlying their pest management decisions, and on pesticide policies and legal regulations at European and national level. The interview guide was designed to structure the communication during the personal interviews, to support the interviewer, and to ensure that all relevant topics were covered. It was reviewed by a group of researchers with different disciplinary backgrounds including agronomy, crop protection, agricultural sociology, and agricultural economics.

The interview guide was organized in six sections and its translation is presented in the Supplementary material (SM1): (i) current weed management on the farm, (ii) experience with and current application of glyphosate-based herbicides, (iii) attitude toward a national glyphosate ban and expected outcomes, (iv) behavioral intention after a potential national glyphosate ban and attitudes toward weed management alternatives if glyphosate-based herbicides were no longer available, (v) facilitating and impeding factors to deal with a national potential glyphosate ban, and (vi) future challenges in farm management beyond a national glyphosate ban. Each section consisted of one to four main, open-ended questions that aimed to encourage interviewees to narrate and to add relevant aspects, where appropriate. Specific sub-questions were adjusted to the respective interview situation, such as the interviewees’ farm types and weed management, and were only asked if an interviewee did not address them when answering the main questions. With respect to weed management, mechanical (e.g., plough), chemical (e.g., alternative selective herbicides), cultivation (e.g., crop rotation), thermal (e.g., flame and steam) and high-tech (e.g., robots) alternatives were addressed. If relevant, topics introduced by the interviewee were picked up by asking additional questions to gain a deeper understanding of the farmers’ perceptions and behavioral intentions. In the interview guide, the topics and questions were carefully arranged to ensure a natural way of talking, and to achieve openness and flexibility (Helfferich 2011; Lamnek and Krell 2016). The sequence and wording of the questions were adjusted to the respective interview situation.

At the end of the personal interviews, each interviewee was asked to fill out a questionnaire about socio-demographic and farm structural data. After the interviews, the interviewer noted further information (e.g., interview atmosphere, interactions between the interviewer and the interviewee, and relevant aspects added by the interviewee) in an interview protocol. A pre-test with three farmers led to minor modifications of the interview guide and the questionnaire.

The interviewer informed each interviewee that participating in the research is voluntary, that he/she will not get paid for participating, that he/she will not suffer from any disadvantage if he/she does not respond to a single or multiple questions, that he/she can stop the interview or withdraw from the research at any time without giving a reason, that he/she is free to contact the research investigator with any questions that may arise, that the collected information is kept confidential, and that direct quotations may be used in publications after being anonymized, i.e., such that the interviewee cannot be identified. All interviewees agreed to contribute to our research and to interview recording. They signed a consent form and received a confidentiality declaration in return.

### Selecting and Recruiting Interviewees

We aimed to include a diverse group of Austrian farms and farmers into our research in order to reveal the range of variations in weed management. More specifically, we followed a purposive sampling strategy by applying the principle of maximal variation (Flick 2014). We chose this strategy because sampling “according to the relevance of cases instead of their representativeness” is “characteristic” for data collection in qualitative research (Flick 2009; p 121). Accordingly, we integrated cases that differ as much as possible with respect to farm characteristics (e.g., farm size, farm type), and regional agricultural production conditions (e.g., soil, topography and climate conditions). We restricted the sample to farmers who had applied glyphosate-based herbicides on their conventionally managed farms at least once within the last five years. This restriction was necessary because of our interest in preferred weed management alternatives if glyphosate-based herbicides were no longer available. It was confirmed by the farm managers at the time of first contact.

For getting in touch with potential interviewees, we combined direct and indirect strategies. With respect to direct strategies, involved researchers relied on their professional networks and searched online for appropriate interview candidates since many farmers run a website. Indirect strategies imply that potential interviewees are suggested by gatekeepers who are authorized to do so. Employees of national and regional agricultural organizations served as gatekeepers (i.e., snowball sampling; Helfferich 2011; Kruse 2014). Following the criterion of theoretical saturation (Glaser and Strauss 2017), sampling was completed when no new information was obtained during the interviews.

### Interview Situation and Characteristics of Interviewees and their Farms

In total, 41 semi-structured interviews were conducted by one interviewer with farm managers across Austria between July and December 2018. Forty interviews were single interviews. One interview was conducted with the farm manager and the adult child who are both working on the farm and both wished to contribute to the interview. The majority of the interviews were carried out on the interviewees’ farms (37). Two interviews took place at the location of the interviewee’s secondary occupation, another one at a neighbor’s farm, and one at the working place of the interviewer. At the time of the interviews, the interviewees were between 24 and 67 years old and mostly male (39). Their farms are located in different agricultural production regions across Austria and vary in size (between 2 and 2739 ha). Furthermore, the interviewed farmers are engaged in different farm types, i.e., field crop incl. field vegetable, permanent crop, grassland and livestock production, and forestry.

The recorded interviews were transcribed word-by-word, whereby personal data were rendered anonymous to maintain confidentiality. The interviewees were coded with the abbreviation “I” for interviewee and a numerical suffix, which indicates the chronological order of the interviews. The codes are used in the result section to trace the quotations. For further information about demographic and farm structural data see Supplementary material (SM2). One interview was not fully recorded and was thus excluded from the empirical analysis. Another four interviews were not considered in the empirical analysis because these interviewees do not apply glyphosate-based herbicides anymore and, thus, do not comply with the defined selection criteria.

### Analyzing the Interviews by Applying the Theoretical Framework

The interview transcripts were systemically analyzed according to the qualitative content analysis by following the content-structuring approach (Mayring 2014; Schreier 2014). Codes were assigned to the descriptive and explanatory information within the transcripts, assisted by the software Atlas.ti (Friese 2019). Deductive and inductive coding was applied, as suggested by Miles et al. (2013). One researcher coded the transcripts in order to achieve consistency. The coding strategy and analytical steps were discussed regularly within the team of researchers. Supplementary details from the questionnaires and interview protocols as well as the interview transcripts from the pre-test were included in the analysis. The latter were considered because modifications during and after the pre-test were only minor.

**Fig. 1:**
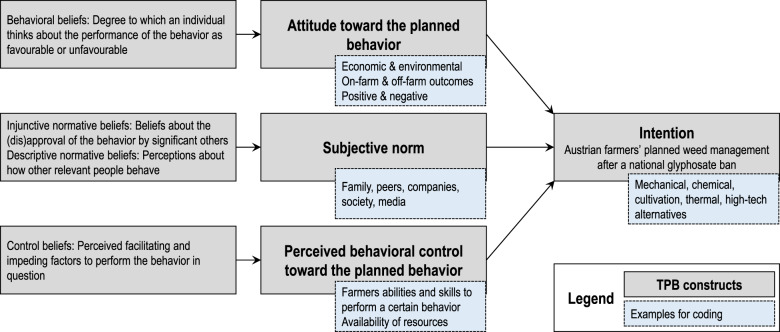
Overview on the theoretical framework used for developing the interview guide, deductive coding, and identifying farmer types in the empirical analysis, based on the Theory of Planned Behavior (Ajzen 1991)

A combined approach of deductive and inductive coding was applied in order to obtain analytical flexibility and to effectively exploit the qualitative data material (Gläser and Laudel 2013; Kuckartz 2014). Deductive codes were defined based on the theoretical framework, i.e., the social-psychological constructs of the TPB (Fig. 1) and the research questions. Deductive codes were applied to the interview transcripts, followed by a careful examination for being appropriate and useful (Miles et al. 2013). If necessary, the codes were revised though the theoretical framework proved adequate. Inductive codes were directly extracted from the empirical text material during the coding process, i.e., already developed codes were refined or subcategorized, and entirely new codes were introduced. Inductive coding allowed us to condense information that goes beyond the social-psychological constructs of the theoretical framework such as factors that indirectly influence behavior.

To give some examples, attitudes toward the intended weed management without glyphosate-based herbicides, subjective norm, perceived behavioral control, and intended weed management after a national glyphosate ban were considered. Intended weed management was categorized into mechanical, chemical, cultivation, thermal, and high-tech alternatives. Attitudes toward the planned behavior and perceived behavioral control were coded for weed management alternatives where possible, while the subjective norm was addressed generally for weed management. Based on the interview material and following suggestions in the literature (e.g., Floress et al. 2017; Reimer et al. 2012; Thompson et al. 2015), economic and environmental attitudes toward the planned behavior were distinguished. On-farm and off-farm outcomes (Mitter et al. 2018) were considered, and evaluative codes (e.g., positive, negative) were used. Subjective norm was coded for important others, such as family, peers, companies from the downstream sector, society and media. With respect to perceived behavioral control, codes included farmers’ abilities and skills to perform a certain behavior, and the availability of resources.

The content-structuring approach was followed by a type-building approach (Mayring 2014; Schreier 2014) in order to allow for comparisons across cases and for identifying patterns of intended weed management. Such a combined approach has been described useful to enhance and condense qualitative research results (Schreier 2014). For type-building, we built on the concept of attribute (or property) space (Barton 1955) and performed four steps (Kluge 1999; Mayring 2014). First, we defined the social-psychological constructs of the TPB as dimensions for type-building and identified the characteristic attributes for each dimension and each individual case (i.e., farmer). Put differently, we used the TPB constructs attitudes toward the behavior, subjective norm and perceived behavioral control toward the behavior to develop the attribute (or property) space, and inductively derived codes to differentiate characteristic attributes. Second, we analyzed empirical regularities and grouped the individual cases along the behavioral intention in question, i.e., farmers’ intended weed management without glyphosate-based herbicides. Third, we defined types by combining the information derived from the previous steps, i.e., we identified types by assigning group-specific characteristics of the TPB constructs to the groups of farmers with similar behavioral intentions. Iterations were necessary to refine the types and assign each individual case to one type. Fourth, we verbally described the defined types of farmers’ intended weed management, which is presented in the next section.

## Farmers’ Intended Weed Management after a National Glyphosate Ban

Mechanical or chemical alternatives—either solely applied or combined with cultivation practices—are the interviewees’ behavioral intentions, given a national glyphosate ban. Thermal weed management and weed management by robots were also addressed during the interviews. However, they are considered immature technologies by all interviewed farmers (Matousek et al. 2019) and are therefore not further discussed.

All interviewed farmers perceive rapid changes in pesticide approvals as challenging and mention a lack of effective alternatives and reduced planning security as major reasons. They disapprove a potential national glyphosate ban because of unfair competitive conditions in a sector where commodities are traded on international markets. Nevertheless, all interviewed farmers recognize the need to change their weed management under a national glyphosate ban and intend to implement mechanical or chemical alternatives, and—where appropriate—in combination with cultivation practices, even if they perceive unfavorable outcomes compared to their current weed management.

With respect to subjective norm, all interviewed farmers perceive a high social pressure to reduce the application of chemical pesticides in general, and glyphosate-based herbicides in particular. Furthermore, almost all interviewed farmers refer to the expectations from actors in the food value chain including processors, retailers, consumers, and interest groups (i.e., injunctive norms). However, most of the interviewed farmers criticize superficial discussions and lacking profundity of the raised arguments. For instance, they complain about media reports focusing on glyphosate-based herbicides because these reports often refer to application practices, which are currently forbidden in Austria (i.e., desiccation).

Almost all farmers see a link between misinformation, lacking knowledge, and the emotional discussions about a glyphosate ban in Austria. However, they comment that negative effects from glyphosate-based herbicides on human health and the environment originate from an incorrect mode of application. Hence, they underline the need for monitoring activities in order to ensure proper herbicide application and use.

We identified four types of farmers who differ in their behavioral intention toward glyphosate-free weed management and the attributes of the TPB constructs. In the following, the four farmer types are characterized along the TPB constructs and major differences are highlighted. An overview is provided in Table 1.

**Table 1:** Overview on the four empirically based types of farmers’ intended weed management after a potential national glyphosate ban, structured along the TPB constructs

	Types of farmers’ intended weed management			
TPB construct	(1) Mechanical alternatives	(2) Mechanical alternatives combined with cultivation practices	(3) Chemical alternatives	(4) Chemical alternatives combined with cultivation practices
Attitude toward the planned behavior	Challenges of a national glyphosate ban perceived small	Challenges of a national glyphosate ban perceived big	Challenges of a national glyphosate ban perceived moderate to big	Challenges of a national glyphosate ban perceived moderate to big
	Overall positive experience, limited on-farm and off-farm outcomes expected	Negative on-farm and off-farm outcomes expected	Negative on-farm and off-farm outcomes expected	Negative on-farm and off-farm outcomes expected
Subjective norm	Negative feedback from media and society on chemical herbicides perceived	Negative feedback from processors, consumers and retailers on glyphosate-based herbicides perceived	Negative feedback from society on chemical herbicides perceived Rejection of mechanically treated fields from peers	Negative feedback from neighbors and society on glyphosate-based herbicides perceived
Perceived behavioral control toward the planned behavior	High level perceived (practical experience and know-how, sufficient equipment)	Low level perceived (insufficient resources)	Low level perceived (unclear about approved chemical alternatives, planning uncertainty)	Moderate level perceived (systemic changes required)

### Farmers’ Intending Mechanical Weed Management Alternatives

This first type of farmers intends to intensify mechanical weed control if glyphosate-based herbicides were no longer available. Farmers belonging to this type classify a national glyphosate ban as a small challenge for their farms because it would require only minor changes in their weed management.

“That’s a small challenge, we’ll somehow manage that mechanically.” (I1)

However, these farmers mostly disapprove a national glyphosate ban, not only because they would like to keep glyphosate-based herbicides for emergency situations, but also because they perceive a national glyphosate ban as unfair in a global agricultural market. Instead of a national ban, they recommend specific application restrictions of glyphosate-based herbicides or a European-wide ban. We assign twelve (out of 36) analyzed interviews to this type of farmers.

Farmers belonging to this type share a positive attitude toward mechanical weed management including plough and cultivator. Mechanical measures are already predominant in their weed management. They prioritize preventive weed control and apply glyphosate-based herbicides rarely and only in small areas. Most of these farmers have consciously reduced the rate of application of glyphosate-based herbicides during the last years and have worked with mechanical alternatives instead. From a national glyphosate ban, they anticipate a necessity for repeated mechanical treatments in order to achieve similar outcomes than with one application of glyphosate-based herbicides. However, they expect limited, though partly negative outcomes for their farms (such as a slight increase in costs and labor time and reduced flexibility) and the environment (such as an increase in CO_2_-emissions from fossil fuel demand) from mechanical weeding. Farmers belonging to this type assess mechanical weed management as manageable because they have gained large practical experience and know-how, they have adjusted farming processes to mechanical measures, are convinced of the effectiveness, and perceive their mechanical equipment as sufficient. The perceived impeding factors are not strong enough to hinder farmers of this type to plan for mechanical measures. Accordingly, these farmers show a high level of control toward their intention, i.e., the application of mechanical measures after a national glyphosate ban.

“Everything where we use chemicals now, we can also do this mechanically. It won’t be much more expensive. We will have to deal with clematis, and we will have to do a bit more manually.” (I25)

By contrast, this type of farmers perceives a lack of chemical alternatives for adequate weed management. They take a negative attitude toward existing chemical alternatives because of expected negative environmental on-farm and off-farm outcomes (e.g., on the soil) and perceive a low control over chemical alternatives (e.g., with respect to usability). These farmers apply glyphosate-based herbicides only in “emergency situations” in order to get rid of persistent weeds and suppress their further sprawl. Others commend its protective character for newly planted permanent crops but perceive its regular application as inordinate. Moreover, these farmers perceive that media and public discussion is emotionally charged against chemical herbicides. Relating to this development, they expect that a national glyphosate ban is followed by an external, national ban of alternative herbicides as well, which would further limit their scope of action. This expectation also contributes to a low control over chemical alternatives and their overall objective to reduce the dependence on chemical herbicides on their farms.

### Farmers Intending Mechanical Weed Management Alternatives Combined with Cultivation Practices

The second type of farmers represents interviewees who intend to combine mechanical alternatives with changes in cultivation, such as changes in crop rotations or crop mixes. These changes would be accompanied by expanding or contractive strategies, i.e., they would either require investments or would lead to a decrease in farm size. Accordingly, these farmers rank a national glyphosate ban as a big challenge for their farms. They are frustrated about the increasing number of banned pesticides, they disapprove the proposed legislative change for glyphosate and emphasize the need for an equivalent alternative. From our interview sample, most farmers (i.e., 16) belong to this type.

Most of these farmers already search proactively for glyphosate alternatives by conducting cultivation experiments on their farms or participating in weed management trainings. Some of them wish for a chemical alternative for emergencies, as they can hardly imagine to get along with mechanical measures solely.

This type of farmers intends to focus on mechanical alternatives, but consider them insufficient for effective farm and weed management. Accordingly, these farmers also intend to reduce direct seeding, cover crops and the cultivation of root crops such as sugar beet. Instead, they intend to return to conventional tillage with repeated mechanical treatments, to switch to mulch-seeding for specific crops, to refrain from cover crops if weather conditions in spring are typically unsuitable for mechanical treatment, and to rise cereal production.

“Sugar beet is over for me, it will no longer be grown then and we are moving toward crops, which you just grow widely, but where’s nothing to earn.” (I11)

These farmers stress negative economic and environmental on-farm and off-farm outcomes of such intended changes. For instance, they evaluate mechanical treatments as more costly and labor-intensive, they expect lower yields, a decrease in soil quality and soil life, and an increase in CO_2_ emissions. The increasing risk of soil erosion by water and wind is particularly emphasized if mechanical weed management replaces direct seeding. Some farmers also refer to likely unfavorable developments for the domestic supply of agricultural commodities.

The farmers assigned to this type perceive their resources including machinery and employees as insufficient for mechanical alternatives, especially during periods of working peaks in spring and summer (i.e., perceived behavioral control). To deal with these perceived limitations, they talk about expanding and contractive strategies. On the one hand, they consider investing in machinery and employing additional seasonal workers. On the other hand, they fear a reduction in farm size or think about more extensive management strategies.

“I have to carefully think about whether I can maintain the size of the farm. […] It will be per hectare, per year 30 more working hours. […] These are working hours that I and my employees cannot achieve, which means I have to invest in a person, I have to invest in a machine.” (I9)

Mechanical alternatives combined with changes in cultivation and resources are the preferred choice under a glyphosate ban despite of the farmers’ undesirable expectations about mechanical alternatives and the perceived limitations in their intentions. However, they rate chemical alternatives as even worse. They underline that approved chemical alternatives are not sufficient for effective weed management. Hence, from chemical alternatives they would expect not only inadequate outcomes with respect to weed control, but also negative outcomes for their business (e.g., increasing costs and labor) and the environment (e.g., higher toxicity of alternative chemicals). Some farmers also fear negative impacts on their health due to negative experiences with the usability of alternative herbicides such as Dicopur. Moreover, these farmers appraise external factors relevant. They suspect further chemical pesticide bans either by law or by food processors and wholesalers which would limit their room for maneuver, in particular, if dependent on chemical herbicides.

### Farmers Intending Chemical Weed Management Alternatives

This type comprises farmers who intend to apply chemical alternatives if glyphosate-based herbicides were no longer available in Austria. They all disapprove a national glyphosate ban and perceive such a ban as a challenge. They assess related changes on their farms as moderate or difficult to achieve. Most of these farmers feel puzzled and unable to cope with such a new situation. Five of the analyzed interviews can be ascribed to this type of farmers.

All farmers belonging to this type intend to combine several selective herbicides and to apply them repeatedly to achieve a similar effect as with one application of glyphosate-based herbicides. From applying herbicide mixes, they expect negative outcomes for their business (e.g., increasing costs) and the environment (e.g., higher toxicity), compared to the application of glyphosate-based herbicides. Furthermore, they consider the relative competitiveness of the domestic farm sector to decrease, and they anticipate increasing imports of cheap agricultural commodities treated with glyphosate-based herbicides. This type of farmers is mostly unclear about appropriate, approved chemical alternatives on the market and perceives a decrease in planning certainty. They all express an urgent need for an equally efficient chemical alternative if the active substance glyphosate is banned. Even though these farmers are aware of the public criticism of chemical herbicides and glyphosate (i.e., injunctive norm), they argue against populistic discussions and classify them as unsubstantiated and biased. In particular, they consider the environmental outcome exerted by weed control on farms with glyphosate-based herbicides as small.

Farmers of this type categorically refuse to apply mechanical alternatives. They share a negative attitude toward mechanical alternatives, because they consider them ineffective or insufficient for weed control. Furthermore, they expect negative economic (e.g., increasing costs, labor and investments) and environmental outcomes (e.g., damages to their permanent crops, decreasing soil quality) for their farms.

“I will certainly not mechanically upgrade to drive through my vine rows every week or 14 days with mechanical machines and permanently damage the vines.” (I30)

The farmers of this type disdain mechanically treated fields from their neighbors or peers (i.e., descriptive norm). Moreover, they perceive a low level of control over using mechanical measures as alternative despite (or because) of their knowledge and experience in using them. On the one hand, they perceive a lack of time, labor and financial resources for mechanical treatments and related new investments. On the other hand, they criticize their low practicability.

“In my opinion, these are jungles. There, the grass is within the middle of the berries – high in the culture. They just didn’t make it. It can really be seen that it is not mechanically manageable.” (I31)

### Farmers Intending Chemical Weed Management Alternatives Combined with Cultivation Practices

This type of farmers intends to focus on chemical alternatives for weed management, given a national glyphosate ban. However, these farmers perceive chemical alternatives as insufficient or only partly compatible with current farm management practices that require them to modify cultivation as well. These intended changes are perceived as challenging by the farmers and, similar to farmers assigned to other types, they ask for an adequate chemical substitute for glyphosate. Based on our qualitative data, we allocate three of the interviewed farmers to this type. The perceived lack of one chemical substitute prompts a mix of several selective herbicides and their repeated application to achieve a similar outcome than with one application of glyphosate-based herbicides.

“I will somehow try a mixture. [laughs] If I’m honest, I don’t even want to bother with the question, but then I’ll try to use other, different active substances.” (I12)

However, expected negative outcomes with respect to the viability of the farm (e.g., increasing costs) and the environment (e.g., increasing toxicity, pesticide residues in soil and water) prevail. Chemical weed control without glyphosate-based herbicides is perceived ineffective for specific crops or in combination with certain agronomic practices including direct seeding, reduced tillage, and other soil conservation practices. Therefore, this type of farmers intends to combine alternative herbicides with additional cultivation practices. For instance, these farmers intend to reduce seed production and root crops such as potatoes and sugar beet especially in areas prone to erosion. Furthermore, they intend to cultivate cropland without cover crops because spraying in spring is perceived more difficult under a glyphosate ban. Farmers engaged in direct marketing intend to increase product prices to cover expected additional costs.

“If I now do a total application with glyphosate, it costs between 10 and 12 € per hectare. [..] If I have to fight grasses, and I spray with a Focus Ultra and afterwards with Dicopur or something else, then I’m about 100 to 150 € per hectare for sure. So there I have a difference of 100 € and I don’t see any improvement for the environment. Absolutely not.” (I19)

Farmers of this type are frustrated from an emotionally driven discussion about a national glyphosate ban and are unhappy about the negative feedback from neighbors and society (i.e., injunctive norm). Therefore, some of them engage in creating awareness toward the relevance of glyphosate-based herbicides for specific applications (e.g., in combination with conservation tillage), for example by commenting on articles in newspapers with letters to the editors.

All farmers belonging to this type (and similar to the farmers represented by the previous type) reject mechanical alternatives because of negative expected outcomes for their business (e.g., increasing costs, decreasing yields) and the environment (e.g., increasing risk of soil degradation, increasing CO_2_ emissions). In particular, farmers who are currently engaged in conservation tillage and focus on humus formation to prevent soil erosion and increase the water-holding capacity of the soil refuse ploughing and repeated mechanical treatments as an alternative. They are not aware of mechanical alternatives that are effective in weed management and, at the same time, do not jeopardize the success of long-term soil improvement practices.

## Discussion

### Factors Influencing Farmers’ Intended Weed Management after a Potential National Glyphosate Ban

The results of our qualitative interviews reveal that farmers intend to respond differently to a potential national glyphosate ban and that various factors influence their intended weed management. The planned responses are either mechanical or chemical alternatives, which would be partly accompanied by changes in cultivation.

Farmers’ attitudes toward the planned response largely affect their intentions. Attitudes are formed based on the evaluation of likely outcomes of a certain weed management alternative, whereby mostly negative economic and environmental outcomes for their farms and for society are perceived relevant. Economic assessments of a potential glyphosate ban mostly confirm unfavorable on-farm outcomes such as reductions in yields and net profits, and increases in total weed control costs and energy consumption (Böcker et al. 2018, 2020). However, they also indicate that aggregate economic outcomes may be small (Böcker et al. 2018; Mitter et al. 2019), despite large variations between crops and regions (Schmitz and Garvert 2012). Effects of the active substance glyphosate on non-targeted plants, animals, microorganisms, and humans have received close attention in recent years (Richmond 2018; Van Bruggen et al. 2018), and environmental on-farm effects have mostly been discussed with respect to potential risks for soil health and erosion (e.g., Silva et al. 2018). However, some interviewed farmers associate environmental outcomes of a new agronomic practice with direct or indirect impacts on their business. For instance, crop farmers link increasing problems with soil erosion due to mechanical weed management and related reductions in soil quality to decreasing yields and profits, especially in the long run. Some farmers are also concerned about the chemical alternatives because of their potentially higher toxicity and negative health impact. The Environmental Impact Quotient (EIQ) was developed to respond to such concerns and guide farmers in comparing environmental impacts of pesticides, whereby “environmental” refers to impacts on farmers, consumers and ecology (Eshenaur et al. 2021; Kovach et al. 1992). EIQ values are regularly updated and provided for a large number of pesticides. For instance, the calculated EIQ value is 15.33 for glyphosate (trade name: Roundup) and 17.41 for dichlorprop (trade name: Dicopur, which was mentioned by the interviewed farmers in this context), meaning that environmental toxicity of a given weight of dichlorprop is indeed higher than that of glyphosate (Eshenaur et al. 2021). The interviewed farmers focus on effective weed management under new regulatory requirements. However, expected outcomes also depend on current weed management adjusted to production conditions and endowments on the individual farms. Farmers who already focus on mechanical weed management and own or have easy access to adequate machinery are convinced of its effectiveness and share both a positive attitude and a high level of control toward mechanical alternatives (as in our identified type one). In contrast, farmers who perceive a high risk of erosion on their fields (because of unfavorable topographic or climate conditions) and currently apply conservation tillage in combination with glyphosate-based herbicides expect strong negative outcomes from mechanical weed management alternatives. These findings are also supported by previous studies, which emphasize that conservation tillage in its current form is largely depending on glyphosate-based herbicides (e.g., Kehlenbeck et al. 2015; von Kröcher et al. 2018). Interestingly, farmers who perceive soil degradation and erosion as a major challenge because their farms are located in a hilly landscape with strong winds or intensive rainfall events intend to apply either mechanical or chemical alternatives, but only in combination with changes in cultivation. While these farmers concede that they would need to change agronomic practices (e.g., reduction in direct seeding) and land use (i.e., cultivated crops) on erosion-prone cropland, production conditions are not decisive whether mechanical or chemical alternatives are intended. This choice seems to depend rather on farmers’ attitude toward the alternatives and their control beliefs (as described for our identified types two and four).

Farmers’ intended weed management is also influenced by subjective norms. Almost all interviewed farmers perceive a low acceptance of chemical measures by society (i.e., injunctive norm), which becomes evident through direct (e.g., walkers passing by the field) or indirect complaints (e.g., via the mayor’s office or controversial discussion on the media). It hampers the intention to apply chemical alternatives even though most farmers criticize the societal refusal of chemical measures because of generally high production standards, comprehensive approval processes in the EU, and restrictions in glyphosate use in Austria (i.e., desiccation). Accordingly, most farmers do not want to bow to that social pressure and perceive a national glyphosate ban as unreasonable. They do not accept societal concerns and call for awareness raising and a factual discussion about a national glyphosate ban instead. Similarly, Kropf et al. (2020) outline that farmers—whether applying chemical pesticides or not—perceive low societal acceptance which reduces their level of control to realize this alternative. Descriptive norms are expressed when individual farmers refer to their colleagues’ farmland that is regularly treated with mechanical alternatives. They perceive organic matter losses because of more intensive tillage and observe that fields are overrun with weeds if chemical pesticides are avoided. This result suggests that the interviewed farmers do not necessarily follow their colleague-farmers’ weed management choices that indicates that they feel low pressure from important others. However, peer farmers were not distinguished (e.g., neighbors running conventional or organic farms, peer-to-peer network) in the interviews and the analysis, while such a distinction could reveal more detailed and potentially different results (see, e.g., Bakker et al. 2021).

Farmers’ control beliefs are also important for their weed management intentions. For instance, farmers are encouraged to apply mechanical alternatives if they perceive their know-how, experience, and equipment as sufficient (as in our identified type one), whereas others are discouraged and intend chemical alternatives because of a perceived lack in financial, mechanical and human resources (as in our identified type three). This conforms to the TPB and is supported by other empirical studies in pest management, which identify limited capacity as a major barrier for reducing chemical pesticide inputs (Bakker et al. 2021). Similarly, Wang et al. (2018) find a large effect of farmers’ control beliefs on their intentions to comply with application standards of pesticides. Moreover, strict regulations, high production standards set by public authorities or private enterprises, and the non-availability of effective chemical alternatives were mentioned (e.g., by our identified type two) to limit behavioral intention toward chemical alternatives. These concerns are also raised by Hillocks (2012) who concludes that future planning with chemical alternatives is fraught with uncertainty, based on an EU pesticide review.

### Applicability of the Applied Theory and Methods

We adopted a qualitative research approach to examine farmers’ intended weed management if the active substance glyphosate was no longer available and to explore which factors, i.e., attitudes toward the planned behavior, subjective norms, and perceived behavioral control toward the planned behavior affect farmers’ behavioral intentions. The interview sample was limited to farmers who produce conventionally and have applied glyphosate-based herbicides at least once within the last 5 years. This allowed us to study the range of planned responses under a potential national glyphosate ban and answer the research questions. However, we also faced some challenges during the research process. First, the recruitment of interviewees was effortful because many farmers were skeptical about the interview topic and expected to be blamed for using glyphosate-based herbicides. Some of them even sent a statement to underline their proper application of glyphosate-based herbicides and only agreed to the interview request if not being condemned. Accordingly, different strategies were pursued to build trust, to establish an impartial interview setting, and to ensure confidentiality. Gatekeepers (e.g., trusted persons in agricultural organizations) and snowball sampling (e.g., interviewees provided access to colleagues) were the most prominent recruitment strategies. Furthermore, the face-to-face interviews, which were mostly conducted in the farmers’ familiar environment, contributed to a pleasant and trustful conversation atmosphere, and the interviewed farmers finally appreciated to talk about their perceptions, fears and behavioral intentions. Personal interviews are also recommended in the literature to reduce potential distance, in particular when critically discussed topics are addressed (Gillham 2005; Irvine 2011).

Second, our empirical research was guided by the TPB, and we investigated farmers’ intended weed management assuming a national glyphosate ban. Behavioral intentions have been shown to be influenced by behavioral, normative and control beliefs, but these may change over time (Ajzen 1991, 2020). Hence, the intention–behavior correlation may decrease with a long time span between the qualitative interviews and an actual ban of the active substance glyphosate. However, we would expect that the major response strategies, i.e., mechanical or chemical alternatives, partly in combination with changes in cultivation, will remain similar if progress in chemical, thermal and high-tech weed management alternatives is slow. Farmers’ reasoning for preferred or intended weed management, however, may change.

Third, our research approach does not enable a prioritization of factors influencing behavioral intentions. Such a prioritization could support the advancement of weed management alternatives to be adjusted to farmers’ individual needs. A quantitative, standardized follow-up survey could provide this information, whereby items could be derived from our qualitative interview material. The combination of qualitative interviews and a quantitative, standardized survey has proven useful for various topics, including climate change perception (Niles and Mueller 2016), climate change adaptation (Rogers et al. 2012), and soil conservation (Bijttebier et al. 2018). Furthermore, studying farmers who already work without glyphosate-based herbicides could provide additional information and inform development, improvement and application of alternatives.

## Conclusions

The political target of reducing the overall use of and risk from chemical pesticides by 50% until 2030, as formulated in the EU 2030 Biodiversity Strategy (European Commission 2020), calls for an improved understanding of farmers’ pest management and how they intend to deal with changes in the policy landscape. Hence, our qualitative research aims to (i) investigate farmers’ intended weed management after a potential national glyphosate ban, (ii) explore factors that influence farmers’ weed management intentions, and (iii) identify types of farmers based on patterns of their behavioral intentions and explored influencing factors. The empirical analysis builds on the TPB and provides a differentiated and detailed understanding of Austrian farmers’ intentions to respond to a discussed national glyphosate ban. We find four types of farmers who intend to choose mechanical or chemical alternatives, either solely or in combination with cultivation practices if glyphosate-based herbicides were no longer available. Major influencing factors of farmers’ intentions are their awareness of adequate weed management alternatives, their appraisal of expected outcomes, and whether they perceive their skills, abilities and resources sufficient for the successful implementation of an alternative. While attitude toward the planned behavior and control beliefs are important for farmers’ intention toward rather mechanical or chemical alternatives, partly combined with cultivation practices, normative beliefs seem to vary only slightly between the interviewed farmers. All interviewees perceive societal disapproval of glyphosate-based herbicides. They desire factual instead of emotional discussions, emphasize the need for adequate substitutes for the active substance glyphosate with similar effectiveness and costs, and would rather accept a European than a national glyphosate ban. These findings suggest that policy instruments to support reductions in chemical pesticide use should focus on behavioral and control beliefs, consider the diversity of influencing factors, and provide know-how on elaborated and sufficiently tested alternatives. However, the design and implementation of successful policies is complex and costly and can be considered as a next research step. Finally, we suggest to investigate the actual behavior of farmers if glyphosate is banned in Austria in order to learn more about it and why intended and actual weed management may differ.

## Supplementary Information

04_SM1_Interview guide

04_SM2_Farm and farmer information
