# Supplementary material for: Farmers’ Intended Weed Management after a Potential Glyphosate Ban in Austria
Source: Environ Manage. 2022 Feb 25;69(5):871–86. doi: 10.1007/s00267-022-01611-0 (PMC9038867; doi:10.1007/s00267-022-01611-0)
Supplement: Supplementary file 1 — 04_SM1_Interview guide [file 267_2022_1611_MOESM1_ESM.docx]

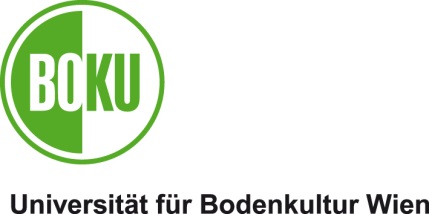
SM1. Interview Guide (translated from German)

| Introduction |
| --- |
| **Acknowledgment** of participating in the study and of taking the time for the interview. |
| **Content introduction:**   - Summary of the **research aims.** - Open questions with focus on **individual perceptions, experiences and expectations.** |
| **Procedure introduction:**   - Ask for **permission** for using the data for research purposes and for recording the interview. - Guarantee of **confidentiality** and **data protection**. - Expected **duration** of the interview: **1 hour.** |
| Ask for **open questions.** |

|  | Guiding questions | Sub-questions | Notes |
| --- | --- | --- | --- |
| **1 Current weed management** | 1.1 When cultivating agricultural land and forests you are confronted with weeds. Could you please tell me how you manage weeds on your farm? | If you think of your crop land/ vineyards/ orchards/ grassland/ pastures/ vegetable production/ Christmas trees/ forests, how do you deal with weeds? | List of glyphosate-based herbicides authorized and available for professional use in Austria in 2018 (36 in total; BAES, 2018).  Barbarian Super 360, Boom efekt, Chikara Duo, Clinic Free, Clinic TF, Dominator Ultra, Durano, Gallup Biograde 360, Glyfos, Glyfos Dakar, Glyfos Envision, Glypho-Rapid 450, Glyphoxx 360 TF, Helosate 450 TF, Kyleo, Landmaster 360 TF, Nasa, Quex Unkrautfrei Premium, Resolva Weedkiller, Rosate Clean 360, Roundup 60, Roundup Alphee, Roundup Easy, Roundup Gel, Roundup Gel Max, Roundup LB Plus, Roundup PowerFlex, Roundup Speed, Roundup Spezial, Roundup Ultra, Roundup Universal, Shyfo, Taifun forte, Technolit Glyphosat 360, Touchdown Quattro, Unkraut-Entferner, Vorox Gierschfrei, Vorox Unkrautfrei Direkt, Vorox Unkrautfrei Direkt AF |
|  | 1.2 You have already mentioned chemical weed management. / You have not mentioned chemical weed management yet. I would like to go into more detail in this topic. Could you please tell me, which herbicide(s) you use in which situation? | If you think of your entire farm, where else do you use herbicides? |  |
| **2 Application of glyphosate-based herbicides** | 2.1 Some herbicides contain the active substance glyphosate. You have already mentioned glyphosate. / You have not mentioned glyphosate yet. **When** do you **apply** glyphosate-based herbicides? | At what time during the year do you apply glyphosate-based herbicides?  How do you **prepare** your fields for sowing?  How do you manage you fields after harvesting?  Could you please tell me which glyphosate-based herbicide(s) you apply? |  |
|  | 2.2 Which **experiences** have you made with glyphosate-based herbicides? |  |  |
|  | 2.3 What do you **know** about glyphosate? | What do you **know** about the **mode of action** of glyphosate?  What do you **know** about potential impacts of glyphosate on the **environment**?  If you have any questions about glyphosate, how do you **inform** yourself? |  |
| **3 National glyphosate ban and expected impacts** | 3.1 There are considerable discussions about the application of glyphosate in agriculture. Even though glyphosate has been approved in the EU until December 2022, discussions are ongoing. In particular, the Austrian government considers a national glyphosate ban. What is your opinion on such a potential ban? | How **realistic** do you consider a glyphosate ban in Austria?  What do you think, how would a glyphosate ban in Austria affect the **environment?**  What do you think, how would a glyphosate ban in Austria affect **erosion control?** |  |
|  | 3.2 Let’s assume that glyphosate-based herbicides are **forbidden in Austria**. Which **impacts** would you expect on your farm? | Which impacts do you expect on your **farm organization**?  Which impacts do you expect on your **working time**?  Which impacts do you expect on your **costs**?  Which impacts do you expect on your gross margins?  Which impacts do you expect on your **yields**? |  |
| **4 Attitudes towards weed management alternatives** | 4.1 In case of a glyphosate ban in Austria, how would you change your **farm management?** | You have already talked about a number of alternatives. There are other alternatives which you have not mentioned yet.  Which changes in **cultivation practices** would you make (e.g. crop varieties)?  How would you change your **crop rotation**?  Which **erosion control** measures would you choose?  How would you treat the drive aisles/ terraces/ vine stocks/ etc. on your farm?  What do you think about using **thermal measures**, like flaming, hot water or hot foam on your farm?  Have you already heard of high-tech measures like a **weed robot?** (I brought a picture of such a weed robot.) What do you think about using a weed robot on your farm? | **Alternatives:**  Chemical measures:   - Selective herbicides   Cultivation practices:   - Crop rotation - Time of sowing and harvesting - Crop variety - Irrigation   Thermal measures:   - Flaming - Hot water - Hot foam   Mechanical measures:   - Additional use of machinery (e.g. plough)   Weed robots:  Weed robots are autonomous mobile machines, that are mostly powered by solar energy. They detect weeds with specific sensors. They work either mechanically or chemically depending on the implemented technique (Slaughter et al., 2008). |
|  | 4.2 We have talked about farm management alternatives **without** glyphosate-based herbicides. Which **experiences** have you already made with such alternatives? |  |  |
| **5 Facilitating/ impeding** | 5.1 If glyphosate was banned in Austria, what would you **need** to run your farm successfully? | What kind of **support would you need**?  What may **hinder** you to run your farm successfully? | e.g. policy measure, societal appreciation |
| **6 Future challenges** | 6.1 A glyphosate ban in Austria may be challenging for your farm. If you think about the next **3 years**, what do you consider the **main challenges** for your farm? | How important do you consider these challenges **compared** to a glyphosate ban in Austria? | e.g. climate change, farm succession, market situation |
|  | 6.2 I am done with my questions and I would like to thank you very much for the insights. Would like to **add** anything on the topic of glyphosate? |  |  |
