# Supplementary material for: Farmers’ Intended Weed Management after a Potential Glyphosate Ban in Austria
Source: Environ Manage. 2022 Feb 25;69(5):871–86. doi: 10.1007/s00267-022-01611-0 (PMC9038867; doi:10.1007/s00267-022-01611-0)
Supplement: Supplementary file 2 — 04_SM2_Farm and farmer information [file 267_2022_1611_MOESM2_ESM.docx]

SM2. Demographic information of the interviewees and farm structural data

| **Code*** | **Age (years)**** | **Gender** | **Agricultural production region***** | **Farm type** | **Farm size (ha)** |
| --- | --- | --- | --- | --- | --- |
|  | 1: <30 2: 30 to <40 3: 40 to <50 4: 50 to <60 5: >60 n/s: not specified | f: Female m: Male | 1: Alpine foothills 2: Carinthian Basin 3: Eastern edge of the Alps 4: High Alps 5: Northeastern flatlands and hill country 6: Pre-Alps 7: Southeastern flatlands and hill country 8: Waldviertel and Muehlviertel region  n/s: not specified | Fi: Field crops incl. field vegetables**** Fo: Forestry Gr: Grassland Li: Livestock Pe: Permanent crops, incl. fruit-, wine & Christmas tree production | 1: <35 2: 25 to <50 3: 50 to <100 4: 100 to <500 5: >501 |
| I1 | 3 | m | 5 | Fo, Pe | 1 |
| I2 | 2 | m | n/s | Fo, Pe | 1 |
| I3 | 1 | m | 6 | Pe | 4 |
| I4 | 3 | m | 5 | Fi, Fo, Gr | 4 |
| I5 | 4 | m | 1 | Fo, Pe | 1 |
| I6 | 4 | m | 1 | Fi, Fo, Gr | 3 |
| I7 | 3 | m | 1 | Fi, Fo, Gr | 4 |
| I8 | 5 | m | 1 | Fi, Fo, Gr | 5 |
| I9 | 4 | m | 5 | Pe | 2 |
| I10 | 2 | m | 5 | Fi | 5 |
| I11 | 2 | m | 5 | Fi | 4 |
| I12 | 4 | m | 5 | Fi | 4 |
| *I13* | 3 | m | 5 | Fi, Fo, Pe | 4 |
| I14 | 3 | m | 8 | Fi, Fo | 4 |
| I15 | 5 | m | 6 | Fo, Gr, Pe | 4 |
| I16 | 3 | m | 7 | Fi, Fo | 4 |
| I17 | 3 | m | 7 | Fi, Fo | 4 |
| I18 | 3 | m | 7 | Fo, Pe | 1 |
| I19 | 4 | m | 7 | Fi, Fo, Pe | 3 |
| I20 | 2 | m | 2 | Fi, Gr | 3 |
| I21 | 2 | m | 2 | Fi, Fo, Pe | 4 |
| I22 | 1 | f | 1 | Fi, Fo, Gr, Pe | 4 |
| I23 | 2 | m | 8 | Fi, Fo | 4 |
| *I24* | 3 | m | 5 | Fo, Pe | 1 |
| I25 | 3 | m | 5 | Fo | 5 |
| I26 | 4 | m | 1 | Fi, Fo, Pe | 5 |
| I27 | 5 | m | 5 | Fi, Pe | 1 |
| *I28* | 4 | m | 5 | Pe | 3 |
| I29 | 3 | m | 5 | Fi, Fo, Pe | 5 |
| I30 | n/s | m | 5 | Fi, Pe | 1 |
| I31 | 4 | m | 5 | Pe | 1 |
| I32 | 4 | m | 5 | Pe | 1 |
| I33 | 4 | f | 4 | Fi, Fo, Pe | 1 |
| I34 | 3 | m | 4 | Fi | 1 |
| *I35* | 3 | m | 4 | Fi, Fo, Gr | 1 |
| I36 | 2 | m | 4 | Fi, Fo, Pe | 4 |
| I37 | 2 | m | 4 | Fi, Fo, Gr | 3 |
| I38 | 3 | m | 5 | Fi, Fo, Gr, Pe | 4 |
| I39 | 3 | m | 1 | Pe | 1 |
| *I40* | 5 | m | 1 | Fo, Gr, Pe | 2 |
| I41 | 3 | m | 5 | Fi | 3 |
| *Notes:*  * Interviews excluded from the empirical analysis *in italic.* One interview was excluded because it was not fully recorded. Four interviews were excluded because these farmers do not apply glyphosate-based herbicides anymore.  ** Age at the time of the interview.  *** Agricultural production regions in Austria, following (Wagner, 1990a, 1990b).  **** On one farm, vegetables are not only produced on fields, but also in greenhouses. | | | | | |
